# Multimodality Characterization of the Clot in Acute Stroke

**DOI:** 10.3389/fneur.2021.760148

**Published:** 2021-12-14

**Authors:** Daniela Dumitriu LaGrange, Isabel Wanke, Paolo Machi, Gianmarco Bernava, Maria Vargas, Daniele Botta, Jatta Berberat, Michel Muster, Alexandra Platon, Pierre-Alexandre Poletti, Karl-Olof Lövblad

**Affiliations:** ^1^Division of Diagnostic and Interventional Neuroradiology, Diagnostic Department, HUG Geneva University Hospitals, Genève, Switzerland; ^2^Division of Neuroradiology, Zentrum für Neuroradiologie, Klinik Hirslanden, Zurich, Switzerland; ^3^Swiss Neuroradiology Institute, Zurich, Switzerland; ^4^Division of Neuroradiology, Institute of Diagnostic and Interventional Radiology and Neuroradiology, University of Essen, Essen, Germany; ^5^Division of Radiology, Diagnostic Department, Geneva University Hospitals, Genève, Switzerland; ^6^Division of Neuroradiology, Zentrale Medizinische Dienste, Kantonsspital Aarau, Aarau, Switzerland

**Keywords:** stroke, clot, computed tomography, micro-CT, electron microscopy

## Abstract

**Aim:** Current treatment of occluded cerebral vessels can be done by a variety of endovascular techniques. Sometimes, the clot responds in varying degrees to the treatment chosen. The *Ex vivo* characterization of the clot occluding the arteries in acute ischemic stroke can help in understanding the underlying imaging features obtained from pre-treatment brain scans. For this reason, we explored the potential of microCT when combined with electron microscopy for clot characterization. Results were compared to the clinical CT findings.

**Methods:** 16 patients (9 males, 8 females, age range 54–93 years) who were referred to our institution for acute stroke underwent dual-source CT.

**Results:** Clinical CT clots were seen as either iso or hyperdense. This was corroborated with micro-CT, and electron microscopy can show the detailed composition.

**Conclusion:** MicroCT values can be used as an indicator for red blood cells-rich composition of clots. Meaningful information regarding the clot composition and modalities of embedding along the stent retrievers can be obtained through a combination of microCT and electron microscopy.

## Introduction

Due to recent advances in clinical imaging in acute ischemic stroke (AIS), various imaging approaches are available for practitioners, as such that they can guide the reperfusion treatment (1–3).

Among the underlying imaging features related to the AIS (4, 5), visualization of the clot can be performed in a clinical setting using either magnetic resonance imaging (MRI) (4–6) or computed tomography (CT) scans (4, 6–8). Information such as clot extent (volume or length) (4, 9–11) and clot shape (12) is known to be linked to the treatment outcome. Red blood cell (RBC)-rich clots can be depicted and measured using the blooming artifact with susceptibility-weighted MRI imaging (4, 5), which is an important indicator for the clot amenability to endovascular treatment. In non-contrast enhanced CT (NCCT) images (4) the clot can be directly visualized when it appears as an area of relative high density within a blood vessel, referred to as a hyperdense artery sign (HAS) (4, 6, 7, 13, 14), and is a highly specific—albeit with low sensitivity—indicator of occlusive stroke (4, 13, 15). In addition, the clot density in CT scan, as measured in Hounsfield Units (HU), can be an indication of the clot composition, in terms of red blood cells or fibrin content, with high density of the HAS being related to RBCs rich clots (16). In contrasting enhanced CT images, the clot can be indirectly visualized via the arterial filling defect. Clot permeability, represented by the residual flow grade (17), is associated with arterial recanalization after thrombolysis. The density of the clot could potentially be used to guide treatment choices and/or predict clinical outcome (18–20). For example, a higher density of the HAS is related to a better angiographic outcome after treatment, either by thrombolysis or thrombectomy (16, 18, 21). The advent of artificial intelligence and automated segmentation methods (22) brings to a new level the potential that clot visualization holds for indicating the underlying clot histological features (23), and for enabling the selection of treatment strategy (24, 25). To confirm the causality between the clot imaging features and the treatment outcome, the *ex vivo* characterization, in terms of clot composition, is necessary (26–28). Conventional histopathology is usually employed to gather information whether the clot is red blood cells-rich or fibrin/platelets-rich (29–31), or, more recently, to discover markers for resistance to treatment (32–34). Alternatively, electron microscopy can offer important information on clot organization, composition, and markers of intravital contraction (29). However, the compositional characteristics of the clot, as examined by histopathology or electron microscopy, are not straightforwardly linked to features observed in clinical imaging, since the blood pool around the clot can contribute, in addition to the clot itself, to the density observed in CT scans. Discriminating between the clot itself and the surrounding blood pool can lead to a more accurate interpretation of brain scans. For this reason, it is important to characterize the clot *ex vivo* with imaging techniques similar to those used in clinical setting. Recently, the preparation of analog clots series (35), spanning a wide range of red blood cells, fibrin, and platelets content, allowed the development of parametric studies which identified MRI sequences (36) and CT protocols (37) capable of differentiating different clot types *in vitro*.

However, clots extracted from patients differ in size, heterogeneity, and compactness from the *in vitro* clots. Characterizing the clots extracted from patients with acute ischemic stroke can be important for understanding how the clot bio-physical properties relate to clinical imaging features, and how such features can be relevant for the diagnosis and treatment of AIS (29). Such understanding will render clinical imaging useful for instituting personalized treatment. The aim of our study is to examine clots that were extracted from patients with acute stroke and examine, with high-resolution techniques, if more information could be obtained about the clot composition. We also aimed to compare, in a pilot experiment, the characterization of the clots in relation to clinical imaging data. Such understanding will, in perspective, render clinical imaging useful for designing personalized treatment.

## Methods

The study has been accepted by our local Ethics Committee (CCER number 2018-00476).

For clot characterization in relation to clinical CT imaging, we included in the study 16 patients (9 males, 8 females, age range 54–93 years) who were referred to our institution for acute stroke, and they underwent dual-source CT in the emergency department, and did not qualify for thrombolytic treatment prior to thrombectomy. Thrombectomy was performed according to the standard clinical practice. For the analysis of the clinical CT scans, the images were uploaded to a computer using OsiriX (Pixmeo, Geneva, Switzerland), and the mean Hounsfield units (HU) values were averaged from at least two regions of interest assigned to the clot occluding the arteries. In addition, with the purpose of examining the clots embedded onto the stents, we included 5 patients (3 males, 2 females, age range 49–87 years, 3 of them received thrombolytic treatment) from which the retrieved clots remained attached onto the stent retriever after thrombectomy.

### MicroCT Imaging

*Ex vivo* experiments were carried out on a low dose X-Ray micro computed tomography scanner (Quantum GX, Perkin Elmer). The scanner uses a cone beam X-ray source and a flat panel X-ray detector to acquire high quality slice images, which are rendered for 3D visualization. CT scans of clots fixed in formalin were acquired on the micro-CT along the maximum intensity projections. The micro-CT can measure the Hounsfield Units (HU) of the analog clots along the x, y, and z axes. Calibration was performed by imaging a water-filled falcon tube, and the HU calibration values were adjusted for air (−1000) and water (0), which allowed measuring the attenuation value of the falcon tube. Then, each clot was rinsed with saline solution, drained on sterile pads, and subsequently placed in a sealed falcon tube to maintain moisture and prevent tissue degradation prior to imaging. The clots were imaged with the low noise imaging protocol (14 min), with 90kV X-ray energy, 88 μA, and 140 μm voxel size. Segmentation and quantification of clots attenuation *ex vivo* was performed in 3D Slicer^1^ (38), using a linear fit, in which air and tube served as reference values.

### Scanning Electron Microscopy (SEM) Imaging

After imaging with microCT, the clots, either self-standing or integrated onto the stent, were fixed in glutaraldehyde (2.5%) overnight at 4°C. Subsequently, samples were washed in phosphate buffer solution (PBS) 10X three times for 20 min each, were dehydrated in solutions of ascending concentrations of ethanol (50, 60, 70, 80, 90, and 100%) for 15 min each time, and were dried using critical point drying. The samples were mounted on scanning electron microscopy (SEM) stubs using carbon tape and carbon paint and sputtered with a 5 nm AuPd (80%/20%) coating. The microscopy observations were performed with an ultra-high-resolution field emission Zeiss Merlin SEM, equipped with a Gemini II column, using the Everhart-Thornley secondary electron detector, 5 kV acceleration voltage and 500 pA probe current.

### Clinical CT Scan Data Analysis

For the analysis of the clinical CT scans, the images were uploaded to a computer using the software OsiriX, and the mean HU values were averaged from at least two regions of interest assigned to the clot occluding the arteries. Analysis was performed by two blinded neuroradiologists who wrote down the numbers.

### Statistical Methods

Hounsfield Units (HU) data is presented in mean ± standard deviation (SD). The normality of data was confirmed using the Shapiro-Wilk test. Two tailed Welch's *t*-test was used to find out if statistically significant differences occurred between the means of fibrin-rich clots and RBCs-rich clots. Receiver-operator characteristic curve (ROC) is used to determine the area under the curve (AUC). All the statistics were calculated using SPSS version 25 (SPSS Inc., Chicago, IL, USA).

## Results and Discussion

Baseline characteristics for patients included in this pilot study, and who did not qualify for thrombolytic therapy, are presented in Table 1. The table includes information on the antithrombotic medication prior to thrombotic event, time elapsed from the thrombotic event (when >4.5 h), conditions contraindicating the thrombolytic therapy, as well as extracted clot characteristics, and thrombectomy outcome.

**Table 1:** Baseline characteristics of patients with stroke included in this pilot study, and which did not receive thrombolytic therapy prior to mechanical thrombectomy.

	Antiplatelet ^*^	Anticoagulant ^*^	Antiplatelet and anticoagulant ^*^	**No antithrombotic medications**
Patients, *n* ^**^	1	9	1	5
Age (years), mean	80	82	71	67
Gender (male), *n*	0	6	1	2
Time thrombotic event onset -to-treatment >4.5 h, *n* (mean, h)	1 (7 h)	2 (8 h)	0	2 (12 h)
**Conditions contraindicating the thrombolytic therapy**, ***n***				
Subcortical hemorrhage	0	0	0	1
Arterial hypertension	1	2	0	2
Intracranial aneurysm	0	0	0	1
Myocardial infarction	0	1	1	0
**Occlusion location**, ***n***				
M1	1	7	0	4
M2	0	2	1	0
P3	0	0	0	1
**Type of clot**, ***n***				
Fibrin-rich—white ^***^, *n*	0	2	0	1
Fibrin-rich—intermediate ^****^, *n*	0	0	1	3
RBCs rich ^*****^, *n*	1	7	0	1
**Endovascular technique**, ***n***				
Aspiration	0	2	1	0
Stent retriever	0	1	0	0
Combination	1	6	1	5
**No. of passes, mean**	2	1.7	4	3.6
**Final TICI score**, ***n***				
0	0	0	0	2
2b	0	0	0	2
2c	0	1	1	0
3	1	8	0	1

* *Medication received prior to thrombotic event*.

** *n, number of patients*.

*** *Fibrin volumetric content > 95%*.

**** *Fibrin volumetric content >70% and <95%, RBCs volumetric content <30%*.

***** *RBCs volumetric content > 85%*.

### Clinical Imaging

**Figure 1:**
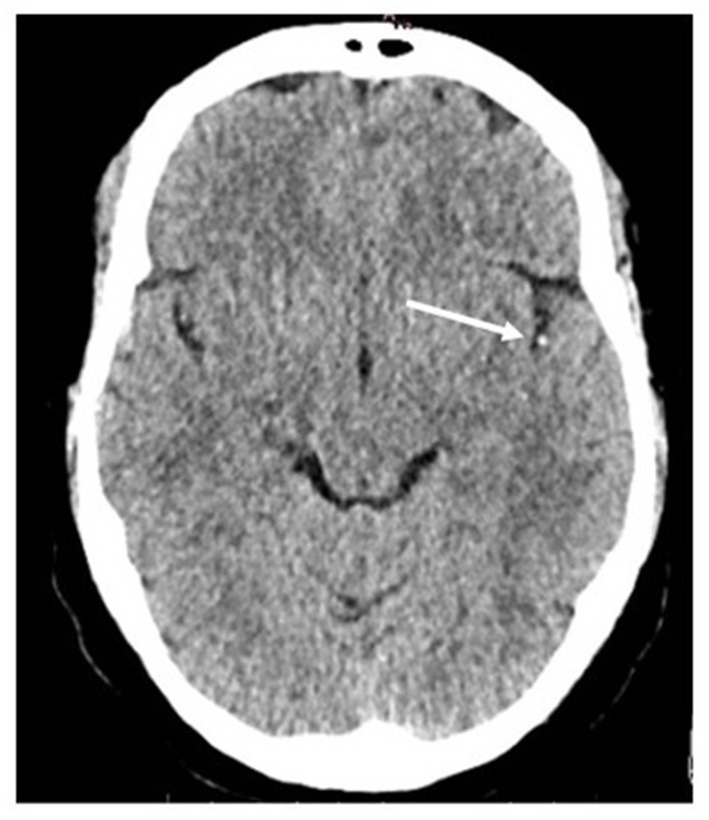
Example of hyperdense artery sign (HAS) in M2 segment occlusion on the left (arrow), in non-contrast CT scan. The extracted clot was a RBCs-rich clot.

**Figure 2:**
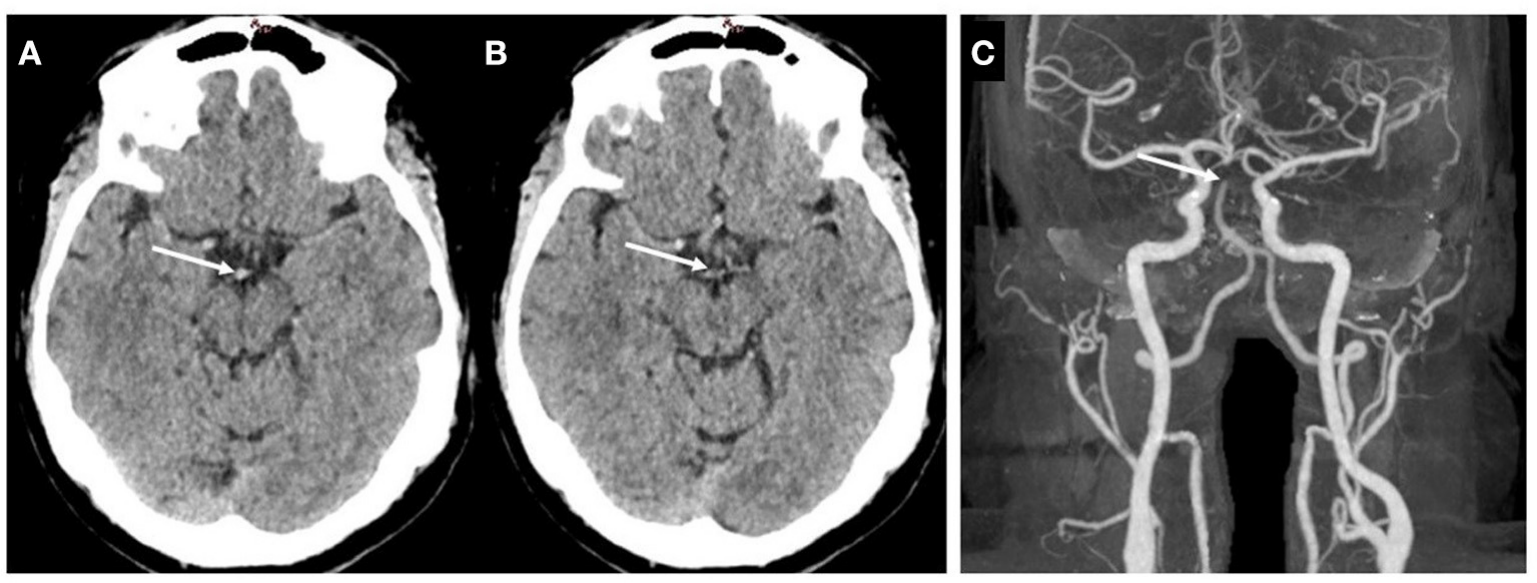
Example of isodense clot. **(A,B)** Non-contrast CT. **(C)** contrast enhanced CT, illustrating the basilar artery tip occlusion. The extracted clot was a fibrin-rich clot.

Typical CT brain scans, in which arterial occlusion sites can be seen as hyperdense or isodense, are illustrated in Figures 1, 2.

### Clots *ex vivo* Examination With Electron Microscopy

**Figure 3:**
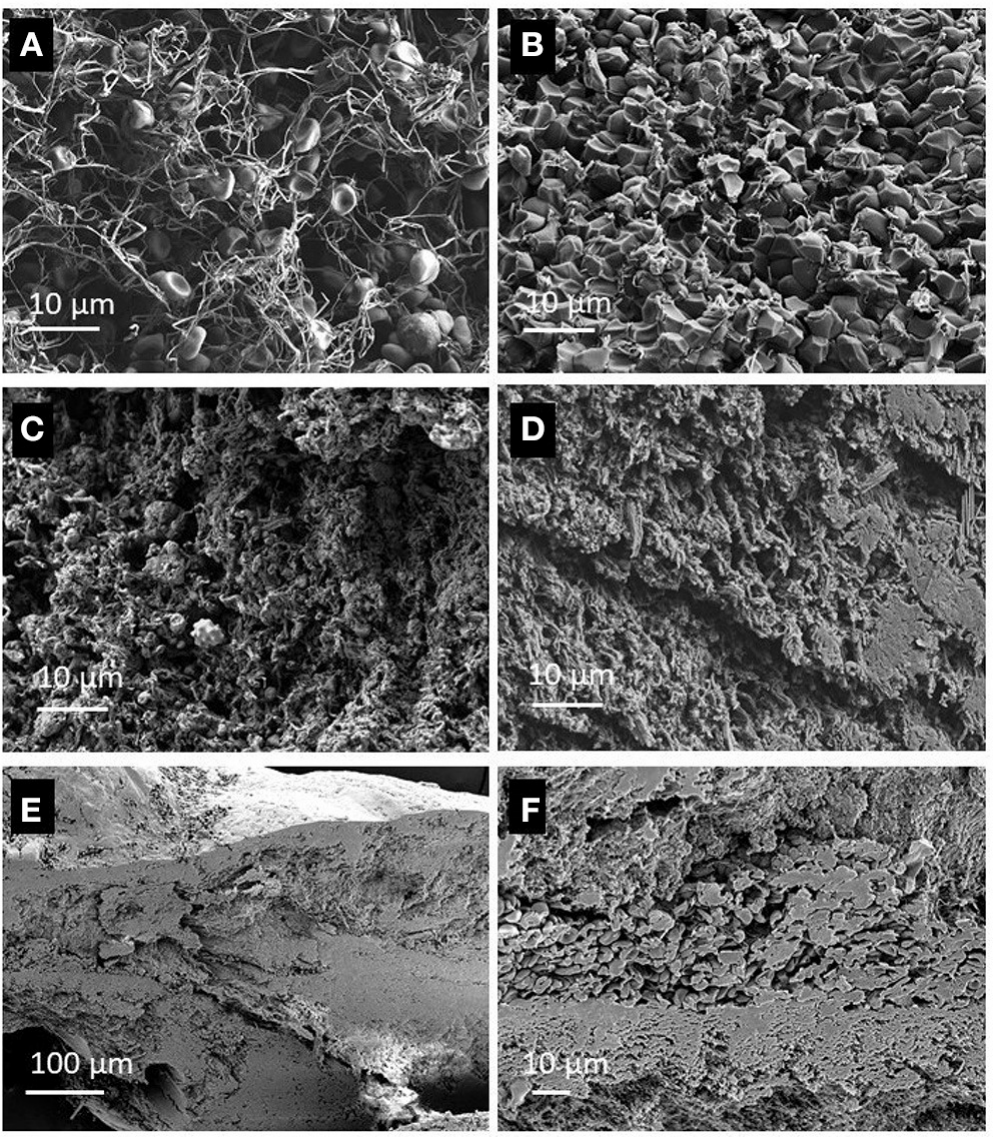
Cross sectional view of clots, recorded with Scanning Electron Microscopy (SEM) showing: **(A)** Periphery of a red blood clot, with biconcave red blood cells in a loose fibrin mesh, **(B)**—The core of the same RBCs-rich clot as in **(A)**, with polyhedrocites as a marker of intravital contraction. **(C)**—A white clot, with fibrin mesh, platelets and white blood cells, **(D)**—Another section of the same white clot as in **(C)**, with dense fibrin and structures with crystalline appearance. **(E)**—A fibrin-rich clot, with scattered red blood cells on the outer surface, and inclusions of biconcave red blood cells, **(F)**—Higher magnification view of the red blood cells and fibrin walls.

Based on our observations with electron microscopy, we categorized the clots as RBC-rich or fibrin-rich. RBC-rich clots are having red blood cells as main volumetric component (>85%), and core regions composed of compact polyhedrally-shaped RBCs (Figures 3A,B). Fibrin-rich clots can have the appearance of a white clot, usually without any red blood cells content (Figures 3C,D) or the appearance of a clot with intermediate composition, in which fibrin remains the main component, as volume fraction, although red blood cells are encapsulated in occasional pits and/or scattered on the outer clot surface (Figures 3E,F). The red clots extracted from patients included in this pilot study have a higher volume compared to the fibrin-rich clots, white or intermediate, and are associated with increased stroke severity, as expressed on the National Institutes of Health Stroke Scale (NIHSS), compared to fibrin-rich white clots, and with better recanalization outcomes compared to fibrin-rich clots (Table 2).

**Table 2:** Extracted clots and clinical features.

	**RBCs-rich clots**	**Fibrin-rich clots—intermediate**	**Fibrin-rich clots—white**
Patients from which the clot was extracted, *n*	9	4	3
Volume of extracted clot (mm^3^), mean (min, max)	48 (18, 110)	24 (12, 30)	8 (4, 15)
NIHSS at admission, mean (min, max)	19 (9, 27)	20 (17, 22)	4 (2,7)
Endovascular treatment, no. of passages, mean (min, max)	2.4 (1, 8)	3.2 (1, 6)	1 (1, 1)
Patients with final TICI score ≥ 2c, *n* (TICI min, TICI max)	9 (2c, 3)	2 (0, 3)	2 (2b, 3)

### Clots *ex vivo* Examination With MicroCT

**Figure 4:**
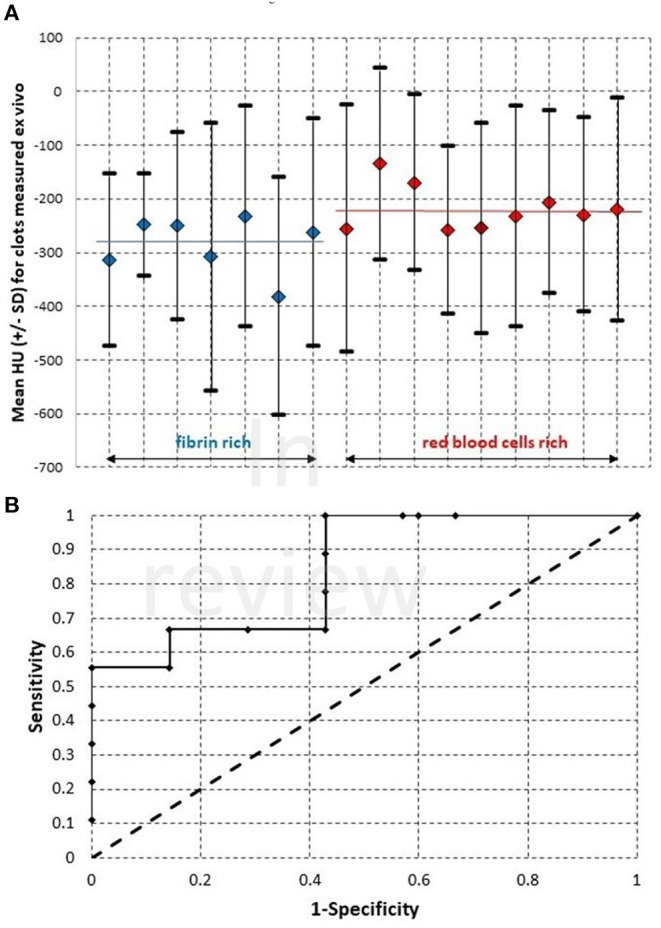
**(A)**—Mean Hounsfield Unit (HU) values and the standard deviation (SD), as measured with microCT for clots extracted from patients by mechanical thrombectomy. The average values for the group of fibrin-rich clots and for the group of red blood cells rich clots are marked with blue bar, respectively red bar on the graph. **(B)**—The ROC curve plotted for the samples shown in **(A)**.

**Figure 5:**
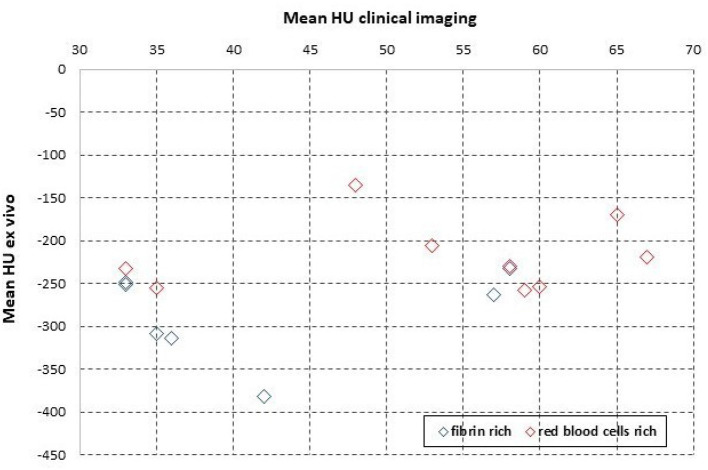
The mean HU values measured *ex vivo* with microCT, for the same samples as in Figure 4, plotted against the values measured in patient, with clinical imaging.

The mean HU values for the clots *ex vivo*, which are obtained from segmenting and quantifying the voxels values with Segment Statistics module in 3D Slicer, were in negative range. SD values are plotted along with the mean HU values in Figure 4A. Using two tailed Welch's *t-*test, a statistically significant difference was found between the means of fibrin-rich clots and RBCs-rich clots, where *t* = −2.784059, and *p* = 0.0173484. The receiver-operator characteristic curve (ROC) is plotted in Figure 4B. Based on these plotted values, the calculated area under the curve (AUC) is 0.84. The mean HU values measured *ex vivo* are plotted against the mean HU values measured in clinical imaging in Figure 5. Using the average value of the mean HU *ex vivo* as cut-off, we calculate a sensitivity of 30% and specificity of 100% for identifying with *ex vivo* microCT the clots that display HAS in clinical imaging. However, no statistically significant association was found between the clots *ex vivo* or those measured on the clinical CT.

### *Ex vivo* Characterization of Clots Embedded on Stent Retrievers

**Figure 6:**
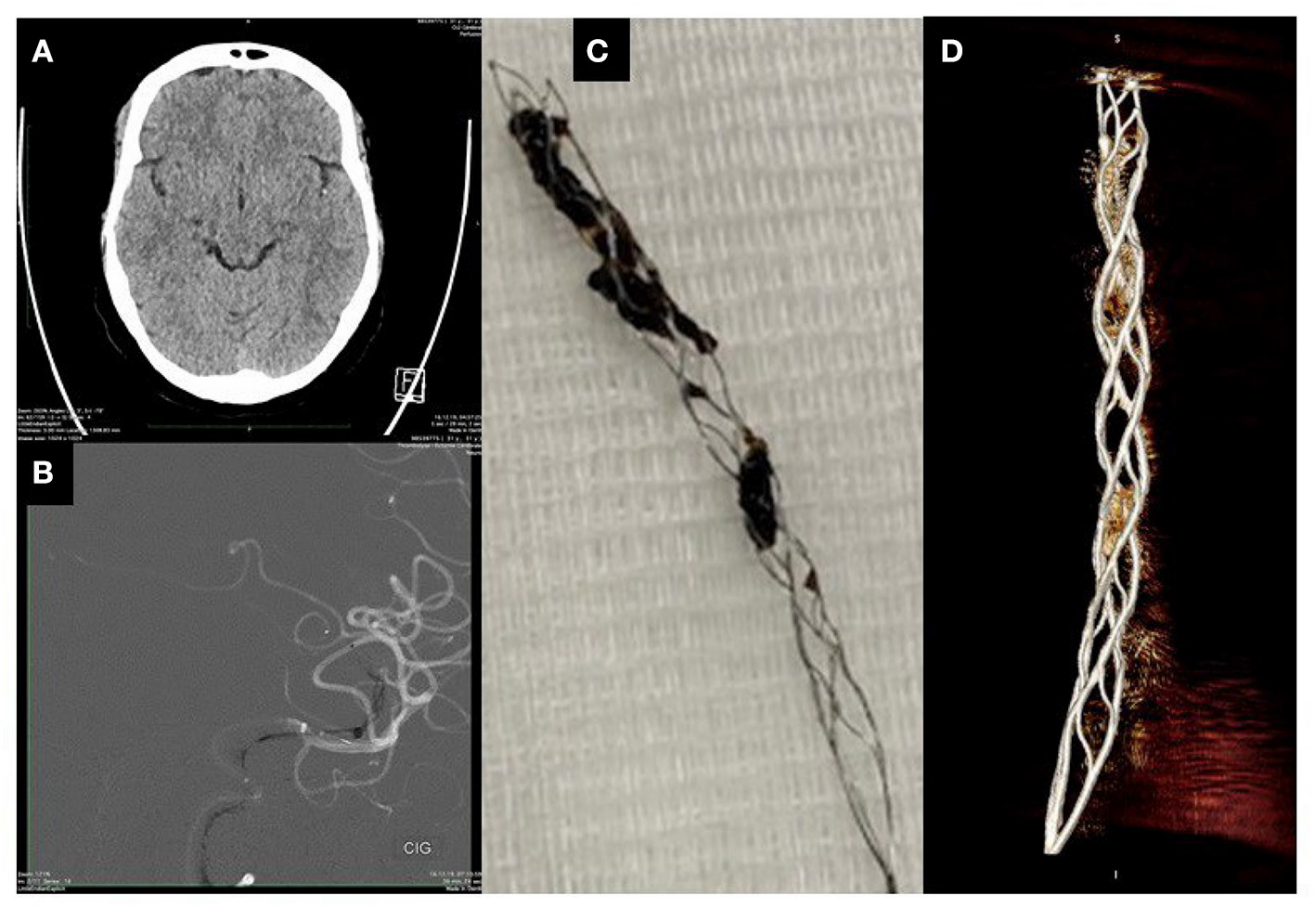
Characterization of a clot on stent: **(A)**—Non-contrast clinical imaging showing HAS, **(B)**—Contrast enhanced CT showing the catheter and the stent deployed at the arterial occlusion site, **(C)**—Optical micrograph of the retrieved clot attached to the stent, **(D)**—MicroCT image of the retrieved clot attached to stent.

**Figure 7:**
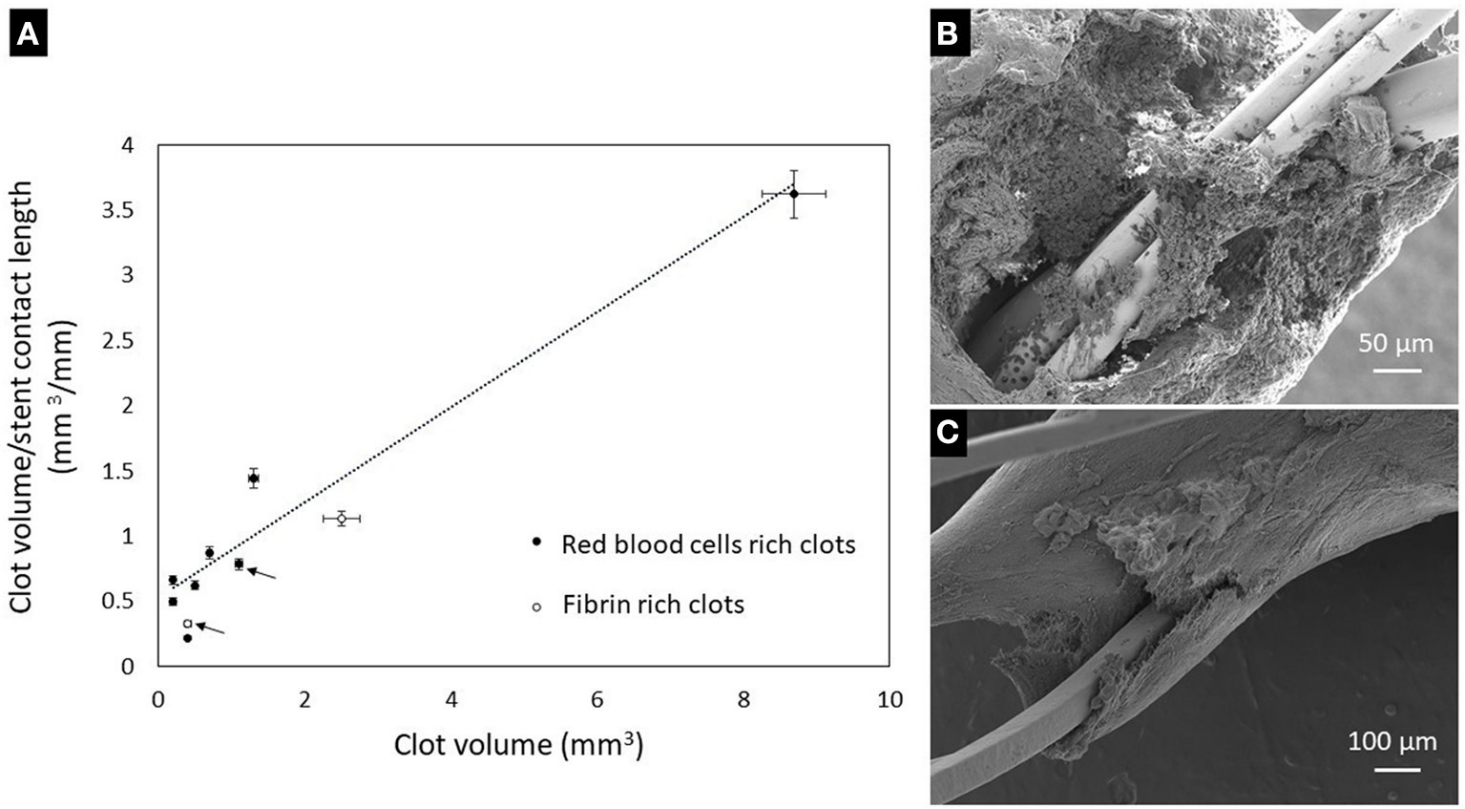
**(A)**—Variation of clot-stent contact surface with the clot volume (the arrows indicate clots retrieved with Solitaire stent retriever, the non-marked clots being retrieved with Trevo stent retrievers). **(B)**—Attachment of a RBCs-rich clot to the stent: the stent struts are protruding through the non-compact region. **(C)**—Attachment of a fibrin-rich clot onto the stent by conforming to the stent strut surface.

We employed microCT along with electron microscopy to characterize the modality through which clots embed along the stent retrievers. A typical microCT depiction of a clot embedded along a stent retriever is illustrated in Figure 6. Both microCT and electron microscopy are useful at identifying the clot volume and the length of the stent covered by the clot. For each patient, we examined the various fragments of clots attached to the stent retriever, and we found that there is a linear correlation (*R*^2^ = 0.9236) between the contact surface (calculated as clot volume-to-stent length covered by clot ratio) and the clot volume (Figure 7A). We found that RBC-rich clots tend to embed through sites at which the stent struts are protruding (Figure 7B). We also found that fibrin-rich clots are embedded along the stent through wrapping around the strut or by wetting the stent surface (Figure 7C). In general, the contact points with the stent are less compact than the core regions, situated between the stent struts.

## Discussion

Characterization of the clot, for example, volume, length (4, 9–11, 39, 40), shape (12), composition, and permeability, are known to be linked to the treatment outcome. More recently, antithrombotic therapy, which is used to prevent thrombus formation, was identified as an important variable in thrombus research (41). In our pilot study, red clots were extracted mainly from patients who received anticoagulant medications prior to the thrombotic event. However, studying the effect of antithrombotic medication onto the thrombus composition can encounter several limitations, and must be cautiously interpreted even when larger sample sizes are studied (41). Polyhedrocites, often found in core regions of red clots, are recognized as markers of intravital contraction (42–44), and potential contributors to stiffness (45). Polyhedrocites were previously found to be the prevailing cell type in red clots, and can be related with clinical features such as stroke severity (44). The findings of our pilot study, mainly focused on patients who did not receive thrombolytic therapy prior to endovascular treatment, are in agreement with previous literature reports (44) and highlight that RBCs-rich clots are more amenable to endovascular treatment, compared to fibrin-rich clots.

Information obtained from clinical data is currently used to make treatment decisions. The composition of the clot, in particular, plays a role in its response to thrombolysis, thrombectomy, and, if recognized in clinical imaging, may even be helpful in deciding which kind of thrombectomy device should be used. It is important to find characterization techniques that better depict clot properties in relation to clinical imaging. In this study, we examined the biophysical properties of the clots in relation to the clinical imaging data. We showed, in a pilot experiment, that clot density, as observed in microCT, is specific for clots with HAS. This finding can be used in designing experiments with larger sample sizes, in which segmentation methods can be used to delineate the clot appearance on clinical CT, and the significance of radiological signs of the clot can be better understood across the various scales. For example, statistically significant associations can be explored between various radiomic features extracted from clinical imaging and those features observed in terms of density (HU) in microCT, along with characteristics related to clot compactness, structure, and composition observed with sub-micron resolution with microscopy techniques. While not yet fully practical, further developments in CT imaging with the use of different scanning techniques, and eventually artificial intelligence, could help determine the clot composition before treatment has started, thereby helping the physician to optimize the choice of therapeutic tools.

## Conclusion

Clots can be successfully imaged at various levels of resolution using microCT and electron microscopy as complementary techniques. Meaningful information regarding the clot composition and modalities of embedding along the stent retrievers can be obtained from these techniques. In perspective, exploration of clots structure and composition with high resolution microCT, using clots in dried state, will improve the sensitivity of this technique. The study of larger sample sizes with high resolution characterization techniques will allow correlative links with clinical imaging, which will be useful for harvesting the underlying information necessary for designing personalized treatment.

## Data Availability Statement

The raw data supporting the conclusions of this article will be made available by the authors, without undue reservation.

## Ethics Statement

The studies involving human participants were reviewed and approved by Swissethics 2018-00476. Written informed consent for participation was not required for this study in accordance with the national legislation and the institutional requirements.

## Author Contributions

DDL, IW, and K-OL were responsible for initiating the project. DDL, JB, and K-OL wrote the manuscript. DDL, PM, DB, MV, and P-AP obtained funding. DDL, IW, PM, MV, JB, and P-AP edited the manuscript substantially. DDL, IW, GB, MM, AP, P-AP, and K-OL collected data. DDL and MM performed studies on samples. All authors contributed to the article and approved the submitted version.

## Funding

The project has been funded by a grant from the Swiss National Science Foundation (32003B_182382) and by a grant from the Radiology Department Startup fund.

## Conflict of Interest

The authors declare that the research was conducted in the absence of any commercial or financial relationships that could be construed as a potential conflict of interest.

## Publisher's Note

All claims expressed in this article are solely those of the authors and do not necessarily represent those of their affiliated organizations, or those of the publisher, the editors and the reviewers. Any product that may be evaluated in this article, or claim that may be made by its manufacturer, is not guaranteed or endorsed by the publisher.

## Footnotes

^1^ http://www.slicer.org
